# Genome evolution in alpine oat-like grasses through homoploid hybridization and polyploidy

**DOI:** 10.1093/aobpla/plw039

**Published:** 2016-07-11

**Authors:** Grit Winterfeld, Alexandra Wölk, Martin Röser

**Affiliations:** Institute of Biology, Martin Luther University Halle-Wittenberg, Neuwerk 21, 06099 Halle, Germany

**Keywords:** Alps, Helictotrichon, hybridization, karyotype, Poaceae, polyploidy, Topoisomerase VI

## Abstract

Hybridization and polyploidization can radically impact genome organization from sequence level to chromosome structure. As a result, often in response to environmental change and species isolation, the development of novel traits can arise and will tend to result in the formation of homoploid or polyploid hybrid species. In this study we focus on evidence of hybridization and polyploidization by ascertaining the species parentage of the endemic alpine *Helictotrichon parlatorei* group. This group comprises five taxa; the diploids *H. parlatorei*, *Helictotrichon setaceum* subsp. *setaceum* and subsp. *petzense*, their putative hybrid *Helictotrichon ×krischae* and the hexaploid *Helictotrichon sempervirens.* For molecular analyses, cloned nuclear Topoisomerase VI genes of *H. sempervirens* and *H.* ×*krischae* were sequenced and compared with sequences of the diploids to estimate the evolutionary history in this group. In addition, detailed chromosome studies were carried out including fluorescence *in situ* hybridization (FISH) with 5S and 45S ribosomal and satellite DNA probes, and fluorochrome staining with chromomycin and DAPI. Two distinct types of Topoisomerase VI sequences were identified. One of them (SET) occurs in both subspecies of *H. setaceum*, the other (PAR) in *H. parlatorei.* Both types were found in *H.* ×*krischae* and *H. sempervirens*. Karyotypes of *H. parlatorei* and *H. setaceum* could be distinguished by chromosomes with a clearly differentiated banding pattern of ribosomal DNAs. Both patterns occurred in the hybrid *H.* ×*krischae*. Hexaploid *H. sempervirens* shares karyotype features with diploid *H. parlatorei*, but lacks the expected chromosome characteristics of *H. setaceum*, possibly an example of beginning diploidization after polyploidization. The geographic origin of the putative parental species and their hybrids and the possible biogeographical spread through the Alps are discussed.

## Abstract (precis)

Molecular cytogenetic and phylogenetic analysis of alpine endemic wild oats of genus *Helictotrichon* represents a remarkable example of speciation and diversification through homoploid hybridisation and polyploidisation. Results suggest a primary centre of species establishment in the eastern regions of the Alps, with the westward expansion and subsequent ice age interruption resulting in a recently disjunct distribution between populations of the southwestern and southeastern Alps.

## Introduction

Grasses, especially the cereals, have long been investigated as model organisms for studies in chromosome organisation, genome dynamics and molecular evolution. The grasses are the most important plant family on earth and represent the fifth largest family in number of species. The systematics and taxonomy of oat-like grasses (tribe Aveneae Dumort.), one of the major groups of grasses, has a long and partly complicated history. Since 1823, when Dumortier (1823) separated the annuals with large and hanging spikelets (*Avena* L. s.str.) from the perennials with more or less small and upright spikelets, many different characters have been used for classification and taxonomy. The instability of the taxonomic treatment of this complex is furthered by the occurrence of intermediate anatomical and morphological characters and combinations thereof, caused by extensive events of hybridization and polyploidization which can lead to newly homoploid or polyploid species (e.g. Matthews *et al.* 2015).

One of the large hybrid-polyploid systems is formed by the perennial oat-like genus *Helictotrichon* Besser, recently newly circumscribed by Wölk and Röser (2013, 2014). In the current valid classification the genus *Helictotrichon* comprises four species complexes distributed in the Mediterranean and the Eurasian mountains, which may have evolutionarily originated in parallel (Röser 1996). One of them is the endemic alpine *Helictotrichon* *parlatorei* group, which forms a clearly defined monophyletic group within the genus (cf. Heuchert and Röser 2004; Röser 1996), sharing a common derived character of diaspores consisting not of single florets, but a compound structure formed by all florets of a spikelet. This is due to a spikelet axis that is not disarticulating at maturity, a character not found in the other taxa of this genus (Röser 1989, 1996). The *H. parlatorei* species complex comprises three species. These are *H. parlatorei* (Woods) Pilg., *H. sempervirens* (Vill.) Pilg. and *Helictotrichon* *setaceum* (Vill.) Henrard with two subspecies *setaceum* and *petzense* (H. Melzer) Röser.

*H.* *parlatorei* is the most widespread taxon of the species group. Based on observations from some isolated populations in the French western Alps, the species seems to be especially abundant mainly along the calcareous mountains of the southern Alps and more scattered in the northern Alps to the southeastern Alps (Karavanke Range, Steiner Alps in Slovenia). However, the species is rare in the dominantly crystalline ranges of the Inner Alps (Aichinger 1933; Gervais 1973; Pignatti 1982 Vollmann 1914). *H.* *parlatorei* is diploid (2*n* = 2*x* = 14) across its distribution (Gervais 1966, 1972; Polatschek 1966; Röser 1989, 1996; Sauer 1971; Winterfeld *et al.* 2009a) except for three tetraploid populations (2*n* = 4*x* = 28) from the Piedmont in the valley of the Chisone River (Gervais 1973; Röser 1989, 1996).

The two subspecies of *H. setaceum* (Vill.) Henrard are distributed in the Southern Alps. The southwesterly distributed subsp. *setaceum* occurs mainly in the French Alps and the Preálpes, but there are additional stands in the Alpes Maritimes. Subspecies *petzense* (H. Melzer) Röser, which was originally described as a separate species, *H. petzense* H. Melzer (Melzer 1967), is distributed on the Petzen and on a few neighboring mountains in the Karavanke Range, the border between Carinthia and Slovenia in the southeastern Alps (Hartl *et al.* 1992; Melzer 1967). Thus, the partial ranges of both subspecies are separated by approximately 600 km. Chromosome numbers of subsp. *setaceum* (Gervais 1965, 1972, 1973; Litardière 1950; Röser 1989) and subsp. *petzense* (Sauer 1971) are diploid (2*n* = 2*x* = 14). The tetraploid number of 2*n* = 28 previously reported for a population of subsp. *setaceum* (Röser 1989, 1996) was due to a sample switching error.

On Petzen Mountain, where *H. setaceum* subsp*. petzense* and *H. parlatorei* grow together, individuals with exactly intermediate morphological characters between these two species occur. These plants have been recognized as a hybrid taxon with *H. setaceum* subsp*. petzense* and *H. parlatorei* as its parental species, named as *Helictotrichon* ×*krischae* by Melzer (1967). The hybrid status was partly confirmed with karyotaxonomical characters by Sauer (1971) and in micromorphological, anatomical and morphometric studies by Heuchert and Röser (2004). In addition to these strictly intermediate hybrids, intermediates between the (F1) hybrids and the parental taxa exist (named *H.* cf. ×*krischae*), displaying a mosaic of morphological characters in the hybrid zone of the Petzen Mountain populations.

The only consistently polyploid taxon of the *H. parlatorei* group, *H. sempervirens* (Vill.) Pilg., is hexaploid with 2*n* = 6*x* = 42 (Gervais 1966, 1973; Litardière 1950; Röser 1989, 1996). It is endemic to the southwestern Alps, namely the French Limestone Alps and their Préalpes, but occurs in only a few stands in the main range of the Alps between France and Italy (Röser 1989, 1996). The distribution of *H. sempervirens* largely corresponds with that of *H. setaceum* subsp. *setaceum*. Both are comparatively rare and only locally frequent (Röser 1989).

In the present study an attempt was made to ascertain the parentage of the *H. parlatorei* species complex by a molecular and cytogenetic survey. Because the commonly used nuclear ribosomal (ITS) or plastid DNA marker sequences (e.g. *matK*) were invariant within this species group (unpublished data), Topoisomerase VI sequences were used for phylogenetic analyses. The single copy nuclear gene Topoisomerase VI shows a highly variable intron structure and seems to be less susceptible to intergenomic concerted evolution. Therefore, the Topoisomerase VI sequences can be used to analyze relationships amongst closely related taxa and offer a suitable resource for phylogenetic studies in polyploids (Brassac *et al.* 2012; Jakob and Blattner 2010; Wölk and Röser 2014).

Combined karyotype studies are the ideal basis for the identification of basic chromosome sets in parental taxa (see Winterfeld and Röser 2007a,b). The present study physically maps the 5S and 45S rDNA as well as two satellite DNAs (CON1, CON2) on chromosomes of the hybrid and proposed parental species using FISH, enabling the detection of hybridization events or chromosomal rearrangements (Abd El-Twab and Kondo 2007; Heslop-Harrison 2000; Jiang and Gill 2006; Kotseruba *et al.* 2003; Mishima *et al.* 2002; Vaio *et al.* 2005). We then map the presence of base pair-specific heterochromatin by differential fluorochrome banding with chromomycin and 4′,6-diamidino-2-phenylindole (DAPI; cf. Schweizer 1976; Schweizer and Ambros 1994; Sumner 1990).

These chromosome mapping methods were used to (i) identify hybrid taxa contributions of the different parental genomes at the DNA sequence and chromosome level, in order to detect a potential continuous transition between *H. setaceum* subsp. *petzense* and *H. parlatorei* and to clarify the origin of polyploid *H. sempervirens*, (ii) obtain insights on chromosomal changes that occurred after hybridization and polyploidization and to assess the role and extent of ‘concerted evolution’, i.e. the homogenization of different DNA repeat types and (iii) reconstruct biogeographical and ecological properties of the different taxa in the hybrid zone of the Petzen and across the entire distribution area in the Alps.

## Methods

### Plant material

Plants were collected in the field from natural populations, either as living plants or as seeds that were later grown in the greenhouse. One sample of each taxon was used for chromosome and molecular survey. Taxon samples and voucher information are shown in Table 1. Vouchers of the accessions studied are housed in the herbarium of the Martin Luther University Halle-Wittenberg (HAL).

**Table 1.** Voucher information of the *Helictotrichon* samples used for chromosome and molecular study, their ploidy level and GenBank accession numbers, collectors (M. Röser, B. Heuchert, G. Winterfeld) and collection number, location of voucher specimens

Taxon	Provenance and collector	Ploidy level	Topoiso-merase 6 clones	GenBank accession number
*H.* ×*krischae* H. Melzer (= *H. parlatorei* × *H. setaceum* subsp. *petzense*)	Austria, Karavanke Mountains; M. Röser 10648 (HAL)	2*x*	KRI_3_	HG797232^b^
			KRI_5_	HG797233^b^
			KRI_7_	HG797234^b^
			KRI_8_	HG797235^b^
*H.* cf. ×*krischae*	Austria, Karavanke Mountains; M. Röser 10649 (HAL)	2*x*		
*H. parlatorei* (Woods) Pilg.	Austria, Karavanke Mountains; M. Röser 10647 (HAL)	2*x*		HG797240^b2^
	Italy, Mt. Baldo; G. Winterfeld 11	2*x*		
	Austria, Karavanke Mountains; B. Heuchert 1108 (HAL)			LN884300
	Italy, Piedmont; M. Röser 2344 (HAL)	4*x*^a^		LN589918^c^
*H. sempervirens* (Vill.) Pilg.	Cultivated in the Botanical Garden of Leipzig	6*x*		
	France, Dépt. Drôme; M. Röser 2429 (HAL)		SEM_1_	HG797243^b^
			SEM_2_	HG797244^b^
			SEM_3_	HG797245^b^
			SEM_4_	HG797246^b^
			SEM_5_	HG797247^b^
			SEM_6_	HG797248^b^
			SEM_7_	HG797249^b^
			SEM_8_	HG797250^b^
			SEM_9_	HG797251^b^
			SEM_10_	HG797252^b^
*H. setaceum* (Vill.) Henrard subsp. *setaceum*	France, Mt. Ventoux; M. Röser 10631 (HAL)	2*x*		
	France, Montagne de Clairet; M. Röser 2420 (HAL)			HG797254^b^
*H. setaceum* subsp*. petzense* (H. Melzer) Röser	Austria, Karavanke Mountains; M. Röser 10646 (HAL)	2*x*		HG797253^b^

a Röser (1989).

b Wölk & Röser (2014).

c Wölk et al. (2015).

### Molecular phylogenetic analyses

#### DNA extraction, amplification and sequencing

Total genomic DNA was isolated from 20 to 45 mg dried leaf material using the NucleoSpin Plant Kit (Macherey-Nagel, Düren, Germany), following the manufacturer’s instructions. The amplified region spans exon 17 to exon 19 of Topoisomerase VI (Topo6), numbered according to the gene structure of the published *Oryza sativa* sequence in EBI (2011). Most Topo6 sequences were amplified using primers Top6-15F and Top6-17R (Jakob and Blattner 2010), binding in exon 17 and exon 19. In some cases, the additional primers TopoHe-18F and TopoHe-18R (Wölk and Röser, 2014), binding in exon 18, were used to amplify the Topo6 region in two fragments. Amplifications were carried out on an Eppendorf Mastercycler (Eppendorf, Hamburg, Germany) using a reaction mix including 2 µl of 10× PCR buffer, 1.9 mM MgCl_2_, 0.8–1 U Taq DNA polymerase (all maximum parsimony (MP) Biomedicals, Heidelberg, Germany), 5% DMSO (AppliChem, Darmstadt, Germany), 0.5 µM of forward and reserve primer, 100 µM dNTPs (GeneCraft, Lüdinghausen, Germany), ∼20 ng template DNA and an aliquot of purified water to obtain a final volume of 20 μl. The PCR program for the amplification using Top6-15F and Top6-17R primers was 3 min at 94 °C, followed by 35 cycles of 30 s at 94 °C, 1 min at 50 °C, 2 min at 72 °C and the final extension at 72 °C for 10 min. We reduced the annealing time of the PCR protocol to 1 min for amplifications using the primer combinations TopoHe-18F/Top6-17R and Top6-15F/TopoHe-18R. PCR products were column-purified with the NucleoSpin Extract II Kit (Macherey-Nagel) and were used for direct sequencing and cloning. The sequencing reaction was carried out with 75 ng DNA and 0.3 µM forward or reverse primer and an aliquot of purified water to obtain a final volume of 15 µl. The reaction mix was sequenced by Eurofins MWG Operon (Ebersberg, Germany).

#### Cloning of PCR products

Cloning of PCR products was carried out using the pGEM-T Easy Vector System of Promega (Mannheim, Germany). Ligation and transformation were performed according to the technical manual. We used the GeneJET Plasmid Miniprep Kit (Fermentas, St Leon-Rot, Germany) to isolate the plasmid, according to the manufacturer’s protocol. Plasmid DNA was eluted in 40 µl nuclease-free water. Presence of the insert was checked by digestion with the restriction enzyme *Eco*RI according to the manufacturer’s protocol (Promega) or by PCR. The restriction product was visualized by gel electrophoresis. The sequencing reaction was carried out by Eurofins.

#### Data and phylogenetic analyses

Sequence editing and the generation of a multiple sequence alignment were both done manually in Sequencher 4.6 (Gene Codes Corporation, Ann Arbor, USA) and ambiguous positions were coded according to the IUPAC Code. Potentially parsimony-informative indels were encoded as binary data and gaps were treated as missing data. We checked rare nucleotide substitutions against the chromatograms to exclude errors in the manual editing. Sequences from *Hordeum marinum* subsp. *gussoneanum* were used as the outgroup according to Wölk *et al.* (2015).

#### Parsimony analyses and bootstrap

A MP analysis was conducted using PAUP* version 4.0b10 (Swofford 2002) and the following options: heuristic search, characters and character-state changes equally weighted, TBR branch swapping, 10 000 maxtrees, RANDOM addition and 100 replications. The heuristic search resulted in several most parsimonious trees, from which a strict consensus was generated. To test the statistical support of the clades a bootstrap analysis with 500 replications, 1000 maxtrees, TBR branch swapping, and closest addition was performed.

#### Bayesian analyses

The best-fit model of nucleotide substitution for the sequence data was performed using MrModeltest 2.3 (Nylander 2004), which selected HKY + G (hLRT) and GTR + G (AIC) as the optimal models for the Topo 6 dataset. We used the more general GTR + G model for the Bayesian inference (BI) analysis, implemented in MrBayes 3.1.2 (Ronquist and Huelsenbeck 2003). The BI analysis was executed for 3 million generations, which were sufficient for an average standard deviation of split frequencies <0.01, with trees sampled every 1000 generations. The first 10% of trees were excluded as burn-in.

### Molecular cytogenetic analyses

#### Chromosome preparation

Young growing root tips were treated in iced water for 24 h, fixed in ethanol:acetic acid (3:1) for 3 h and stored in absolute ethanol at −20 °C. Before preparation the root tips were softened in 1% cellulase (w/v) and 10% pectinase (v/v) in citric acid-sodium citrate buffer pH 4.8 at 37 °C (cf. Schwarzacher *et al.* 1980) and squashed in 45% propionic acid with 2% carmine. After removal of the cover slips by freezing the slides at −90 °C, immersing in absolute ethanol for 5 min and air drying, they were stored at −20 °C until *in situ* hybridization and fluorochrome banding.

#### *DNA probes for* in situ *hybridization*

DNA probes used for *in situ* hybridization were 5S rDNA (GenBank AJ390195; Röser *et al.*, 2001), parts of 45S rDNA (GenBank Z96856, Z96857; Grebenstein *et al.* 1998), satellite DNA CON1 (GenBank Z68761-68765) and satellite DNA CON2 (GenBank Z68772-Z68775; Grebenstein *et al.* 1995, 1996). The probes were labeled with fluorescein-11-dUTP using the ‘random primed labeling kit’ of Roche Diagnostics, according to the manufacturer’s instructions.

#### FISH, fluorochrome banding and signal detection

For detection of the ribosomal and satellite DNA probes, FISH was done successively according to the protocols of Leitch (1994) with modifications of the procedures described in detail in Winterfeld and Röser (2007a). Unhybridized DNA was counterstained with propidium iodide (1.5 mg ml^−^^1^) dissolved in fluorescence antifade solution (Vector Laboratories). Metaphase chromosomes on the slides were screened with a Zeiss Axioskop 2 epifluorescence microscope equipped with filter block 09 for simultaneous detection of fluorescein and propidium iodide fluorescence. To reveal GC or AT-rich chromosome segments by fluorochome banding the slides were cleaned in distilled water, air-dried, and stained with a 0.03% solution of chromomycin A_3_ (Sigma, St Louis, MO, USA) in McIlvaine’s buffer pH 7.0 containing 5 mM MgCl_2_ for 1.5 h before embedding in antifade solution (Vector Laboratories) containing 2 mg ml^−^^1^ DAPI. The slides were stored in the dark until examination by epifluorescence microscopy with the Zeiss filter blocks 05 for chromomycin and 02 for DAPI. Photographs were taken using a computer-assisted cooled CCD camera (Zeiss Axiocam HRC) employing Zeiss Axiovision software.

#### Chromosome analyses and graphical representation

**Figure 1.**
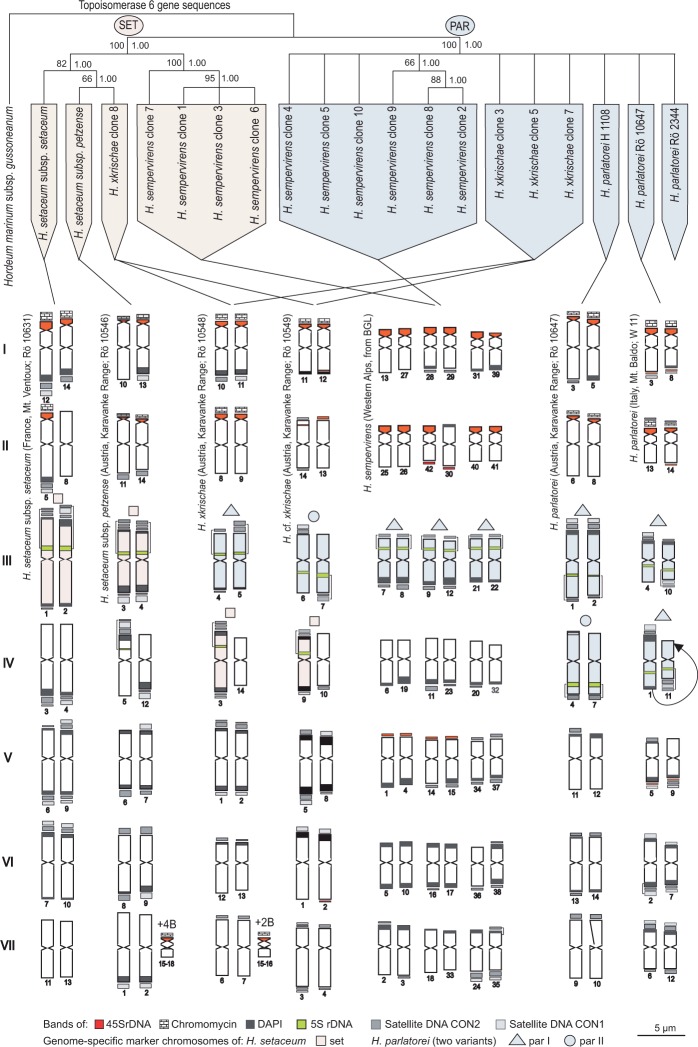
Idiograms of chromosome complements of diploid *Helictotrichon* species and hexaploid *H. sempervirens* on a strict consensus tree derived from Topoisomerase IV gene sequences. Bootstrap and Bayesian support are shown on the left and right side above the branches. Chromosomes are numbered below according to their length and arranged into groups of presumable homologues or homoeologues (lines I–VII) according to FISH with 45S and 5S rDNA probes and fluorochrome banding signals. Chromosome bands arranged above or below the chromosomes are subtelomeric, unless an intercalary position is indicated by a square bracket at the side of the respective chromosome. Chromosomes with a NOR bearing the 45 S rDNA bands or chromomycin-positive staining are indicated by a secondary constriction in the chromosome idiogram additional to their centromeric constriction (= satellite chromosomes; cf. Winterfeld and Röser 2007b). Genome-specific DNA and chromosome variants are shown in light blue for *H. parlatorei* and in pink for *H. setaceum*. Possible mechanisms of chromosome and genome evolution as well as geographical distribution of the species are listed below. B = B chromosomes.

The chromosome sizes, localization of primary constrictions (centromere), secondary constrictions (satellite region; SAT = localization of nucleolus organizer regions, NORs) and extension of chromosome bands (5S rDNA, 45S rDNA, chromomycin and DAPI bands) were measured digitally using Zeiss Axiovision and Adobe PhotoShop 6.0 software. Measurement data was input into Microsoft Excel for calculations. Chromosomes were numbered according to their individual length. Complete chromosome idiograms were constructed using CorelDraw software. In the idiograms the chromosomes were arranged into groups of presumable homologues or homoeologues (indicated by Roman numerals in lines I–VII in Fig. 1) according to FISH and fluorochrome banding signals. Satellite chromosomes representing a secondary constriction, 45S rDNA band or chromomycin positive and DAPI negative bands (cf. Winterfeld and Röser 2007a) were arranged in lines I and II (Fig. 1). The terminology for chromosome shape according to the centromere position (metacentric or submetacentric) followed Levan *et al.* (1964).

## Results

### Molecular phylogenetics

#### DNA-sequences

Due to nucleotide additivity in direct sequencing of Topo6 in 2*x H.* ×*krischae* and 6*x H. sempervirens*, the PCR products of these taxa were cloned and up to ten individual clones were sequenced. The alignment of 20 sequences from direct sequencing (three accessions of *H. parlatorei*, two of *H. setaceum*) and from cloned fragments (*H.* ×*krischae*, *H. sempervirens*) comprised 922 nucleotide positions. 53 of 180 variable characters were parsimony-informative. The sequences obtained from this study are available in GenBank (see Table 1).

#### Partial alignment

In total, 48 of the parsimony-informative nucleotide sequence variants and their positions are shown in the partial alignment depicted in Table 2. Clone 8 of *H.* ×*krischae* and clones 1, 3, 6 and 7 of *H. sempervirens* conform as far as possible (85%) with the sequences of the two subspecies of *H. setaceum* (marked in pink in Table 2). However, five variable nucleotides, 193 (A/T polymorphism), 226 (T/A), 295 (A/G), 384 (T/G) and 650 (A/G), in clone 8 of *H.* ×*krischae* agree with the repeat types of the two subsp. of *H. setaceum* but differ from clones 1, 3, 6 and 7 of *H. sempervirens*. At positions 75 and 76 an additional polymorphism is found (G/A), in which clone 8 of *H.* ×*krischae* matches *H. setaceum* subsp. *petzense*. Clones 3, 5 and 7 of *H.* ×*krischae* and clones 2, 4, 5, 8, 9 and 10 of *H. sempervirens* closely match sequences of *H. parlatorei* (marked in light blue in Table 2). Despite 180 variable characters (point mutations) in the whole alignment (not shown) single base pair changes occur in the partial alignment of Table 2 at positions 15 (clone 3 of *H.* ×*krischae*: A/G polymorphism), 43 (clone 5 of *H.* ×*krischae*: T/- polymorphism), 242 (*H. parlatorei* accession H 1108: G/- polymorphism), 295 (clone 7 of *H. sempervirens*: A/G polymorphism), 429 (clones 4, 5 of *H. sempervirens* and *H. parlatorei* accession M. Röser 2344: T/C polymorphism), and 683 (*H. parlatorei* B. Heuchert 1108: C/- polymorphism).

**Table 2.** Variable nucleotide positions of Topoisomerase 6 in direct sequencing of PCR products in 2*x H. parlatorei* (blue shading), *H. setaceum* subsp. *setaceum* and subsp. *petzense* (red shading) and cloned sequences of 2*x H.* ×*krischae* and 6*x H. sempervirens.*

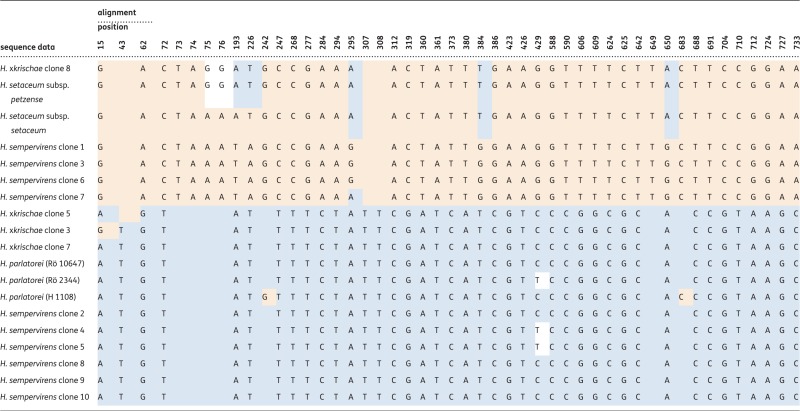

#### Phylogenetic analyses

A heuristic search found a single tree island consisting of 6956 equally parsimonious trees with a length of 201 steps. All trees in the island have a high consistency and retention index (CI = 0.965, RI = 0.9727) which indicates a low level of homoplasy in the data matrix. MP and Bayesian analysis resulted in similar tree topologies. A strict consensus of all most-parsimonious trees is shown in Figure 1, with posterior probability (PP, on the right side above the branches in Fig. 1 values of the Bayesian analysis (BS, on the left side above the branches in Fig. 1). Two clades were maximally supported. The first clade shows support for relatedness of both subspecies of *H. setaceum* and clone 8 of *H.* ×*krischae* (BS 82%, PP 1.00) that are closely related to a strongly supported clade comprising clones 1, 3, 6 and 7 of *H. sempervirens* (BS 100%, PP 1.00; copy type SET; cf. Wölk *et al.* 2015; marked in pink in Fig. 1). The second clade includes all populations of *H. parlatorei* and clones 2, 4, 5, 8, 9 and 10 of *H. sempervirens* (BS 100%, PP 1.00; copy type PAR; marked in light blue in Fig. 1); for the most part relationships among these sequences are unresolved within this clade.

### Molecular cytogenetic analyses

#### Karyotypes

**Figure 2.**
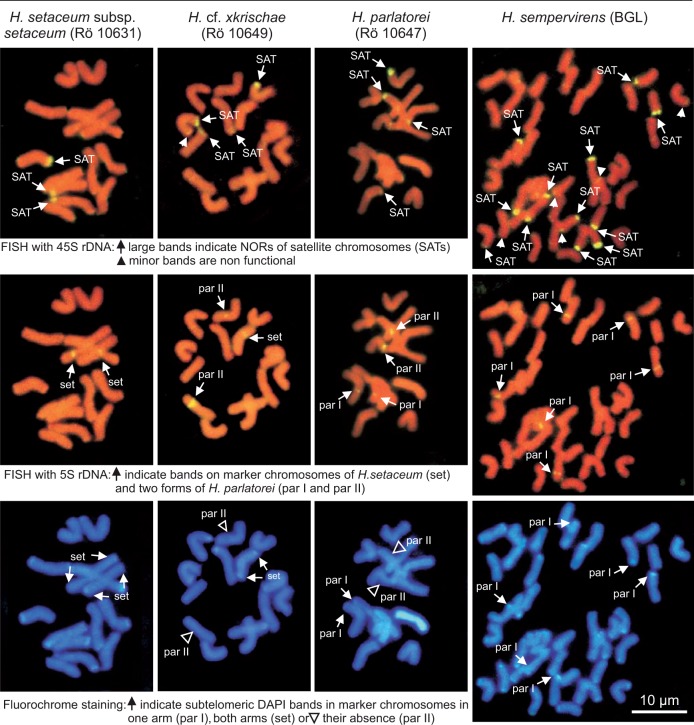
Somatic metaphase plates of *Helictotrichon* species after FISH and fluorochome staining.

Figure 2 shows mitotic metaphases of *H. setaceum*, *H. parlatorei*, *H.* ×*krischae* and *H. sempervirens* after *in situ* hybridization and fluorochome staining. The respective idiograms of all taxa investigated are shown (Fig. 1). The karyotypes are constituted by seven pairs of chromosomes (Roman numerals; Fig. 1) in 2*x H. setaceum*, *H. parlatorei* and *H.* ×*krischae*, and three sets of seven chromosome pairs in 6*x H. sempervirens*. All chromosomes are metacentric with the exception of one pair in *H.* cf. ×*krischae* (chromosomes 3 and 4), which are submetacentric.

#### Satellite chromosomes

In all taxa the presence of four terminal SATs per diploid chromosome complement was observed at the distal end of the short arms identified by secondary constrictions, 45S rDNA bands and/or chromomycin-positive and DAPI-negative bands (Fig. 2). One exception was found in *H. setaceum* subsp. *setaceum*, which displayed only three SATs (obviously loss of one NOR). Additionally, subtelomeric minor 45S rDNA bands were found in *H. parlatorei* (Winterfeld 11; chromosomes 3, 5, 8, 9, 14), *H.* ×*krischae* (chromosomes 2, 12) and *H. sempervirens* (chromosomes 1, 4, 14, 15, 30, 42).

#### 5S rDNA-bearing chromosomes

Bands of 5S rDNA are intercalary in non-satellite chromosomes, either in the long or in the short arms, with two, three or four loci per diploid complement. Two loci occur in chromosomes 1 and 2 of *H. setaceum* subsp. *setaceum* and chromosomes 7, 8, 9, 12, 21 and 22 of *H. sempervirens*. Three loci occur in chromosomes 3, 4 and 5 of *H. setaceum* subsp. *petzense*, chromosomes 3, 4 and 5 of *H.* ×*krischae* and 6, 7 and 9 of *H.* cf. ×*krischae*. Four loci occur in chromosomes 1, 2, 4 and 7 of *H. parlatorei* (M. Röser 10647) and 1, 4, 10 and 11 of *H. parlatorei* (Winterfeld 11).

#### Bands of DAPI positive heterochromatin and satellite DNAs

DAPI-positive bands, if present, are subtelomeric either in both arms or only in one arm. They are often, but not always, correlated with the loci of the satellite DNAs CON1 and CON2.

In the chromosome complement of *H. setaceum* subsp. *setaceum* broad DAPI bands occur in the long arms of all SAT chromosomes (5, 12, 14), in both arms of the 5S rDNA-bearing chromosomes (1, 2), in both arms of two non rDNA-bearing chromosome pairs (chromosomes 6, 7, 9, 10) and in one arm of an additional pair (chromosomes 3, 4). They are correlated mostly (chromosomes 3, 4, 5, etc.) but not always (e.g. chromosomes 7, 10) with the bands of satellite DNAs CON1 and CON2 (Fig. 1). In three chromosomes (8, 11, and 13) no chromosome band was found.

In *H. setaceum* subsp. *petzense* only one of the four satellite chromosomes had a subtelomeric DAPI band (chromosome 13), two showed broad bands of satellite DNA CON2 (11, 14) and one had no DAPI or satellite DNA band. As seen in subsp. *setaceum* the 5S rDNA-bearing chromosomes 3 and 4 had subtelomeric DAPI bands in both arms, which are correlated with bands of the satellite DNAs CON1 and CON2. Additional DAPI bands were found in both arms of chromosomes 6 and 7 and in one arm of chromosomes 1, 2, 5, 8, 9 and 12, partly corresponding with the bands of satellite DNAs CON1 and/or CON2 (Fig. 1).

Karyotype structures and banding pattern in both putative hybrid accessions *H.* ×*krischae* (M. Röser 10548) and *H.* cf. ×*krischae* (M. Röser 10549) were very similar. Two satellite chromosomes showed subtelomeric DAPI bands in the non-satellite arms (chromosomes 10 and 11 in accession M. Röser 10548; 11 and 12 in M. Röser 10549) but the two other SAT chromosomes 8 and 9 (M. Röser 10548) and 13 and 14 (M. Röser 10549) were devoid of DAPI or satellite DNA bands. Two of the 5S rDNA-bearing chromosomes 4 and 5 (M. Röser 10548) had small DAPI bands in only one arm, the non 5S rDNA-bearing arm, while the presumable homoeologous chromosomes 6 and 7 of the accession M. Röser 10549 are devoid of DAPI bands. The third 5S rDNA-bearing chromosome of both accessions had, like both subspecies of *H. setaceum*, DAPI bands in both arms, which are correlated with bands of the satellite DNAs CON1 and/or CON2. In the remaining chromosomes, DAPI bands occurred in both arms (1, 2 in M. Röser 10548; 5, 8 in M. Röser 10549), in only one arm (12, 13 in M. Röser 10548; 1, 2 in M. Röser 10549) or were absent (chromosomes 6, 7, 14 in M. Röser 10548; 3, 4, 10 in M. Röser 10549), which is partly correlated with the bands of the satellite DNAs CON1 and CON2 (see Fig. 1).

In the hexaploid chromosome complement of *H. sempervirens* in four of twelve satellite chromosomes subtelomeric DAPI bands occur in the arms lacking satellites. All 5S rDNA-bearing chromosomes reveal DAPI bands in the arms without the 5S rDNA loci. Five chromosomes (5, 10, 16, 17, 38) had DAPI bands in both arms; two chromosomes were devoid of DAPI bands (14, 36). The remaining chromosomes showed DAPI bands in one arm, partly correlated with the satellite DNAs CON1 and CON2 (Fig. 1).

*H.* *parlatorei* M. Röser 10647 from Petzen Mountain is very similar to the population G. Winterfeld 11 from Monte Baldo with respect to DAPI banding. In both populations, only one of the four satellite chromosomes had DAPI bands near the telomeres (chromosome 5 in M. Röser 10647, 8 in G. Winterfeld 11). Two of four 5S rDNA-bearing chromosomes show DAPI bands in one arm (1, 2 in M. Röser 10647; 4, 10 in G. Winterfeld 11). In other 5S rDNA-bearing chromosomes DAPI bands were absent (4, 7 in M. Röser 10647; 11 in G. Winterfeld 11), while chromosome 1 of G. Winterfeld 11 revealed DAPI bands in both arms. In the remaining chromosomes DAPI bands were localized either in one arm (11, 12, 13, 14 in M. Röser 10647; 5, 6, 9, 12 in G. Winterfeld 11), in both arms (13 in M. Röser 10647; 2, 7 in G. Winterfeld 11) or were absent (9, 10 in M. Röser 10647).

## Discussion

### Structures of parental genomes at sequence and chromosomal level reveal hybrid origins: biogeography and evolution of the *H.* *Parlatorei* group

#### Hybrid evidence at sequence level

Sequence variation in the nuclear single copy gene Topo6 and resulting trees show the occurrence of two different copies, in taxa of the *H. parlatorei* group (SET and PAR, Fig. 1; Wölk *et al.* 2015). In the diploid taxa, SET type copies occur in both subspecies of *H. setaceum*, whereas PAR type copies were found the three populations of *H. parlatorei* investigated (see Table 2). Both the diploid hybrid populations of *H.* ×*krischae* and *H.* cf. ×*krischae* and hexaploid *H. sempervirens* have two distinct variants of Topo6 sequences, which correspond to SET and PAR, respectively. The resulting gene phylogeny (Fig. 1) corroborates the hybrid character of *H.* ×*krischae* and *H.* cf. ×*krischae*, in accordance with the systematic treatment based on morphological and anatomical characters by Melzer (1967), the classical karyological investigation by Sauer (1971) and micromorphological, anatomical and morphometric studies by Heuchert and Röser (2004). Hexaploid *H. sempervirens* is shown to be an allopolyploid hybrid species between *H. setaceum* and *H. parlatorei* due to the occurrence of their two Topo6 gene copies.

#### Detection of marker chromosomes and reconstructing of hybrid origins

The presence of four satellite chromosomes per 2*n* = 2*x* = 14 chromosomes is a consistent karyotype found in the *H. parlatorei* group diploids. The only exception is *H. setaceum* subsp. *setaceum* with only three satellite chromosomes, most likely due to the loss of one NOR in the specimen sampled. However, karyotypes of *H. parlatorei* and *H. setaceum* are distinguished by differences in the amount and distribution of heterochromatin indicated by DAPI bands and also by bands of the satellite DNA s CON1 and CON2 (cf. Winterfeld and Röser 2007b), especially in the 5S rDNA-bearing and the SAT chromosomes. The occurrence and localization of the heterochomatin bands in 5S rDNA-bearing chromosomes provide the identification of parental species marker chromosomes involved in the formation of hybrid taxa between *H. parlatorei* and *H. setaceum*.

The karyotypes of the two populations of *H. parlatorei* studied are very similar, characterized by the occurrence of two chromosome pairs with intercalary 5S rDNA bands. These pairs occur in two slightly different variants; with small subtelomeric bands of satellite DNAs and DAPI in the arm without the 5S rDNA, termed marker chromosome par I (light blue triangle in Fig. 1), or without additional bands, termed par II (light blue circle in Fig. 1). The two DAPI bands in both arms of chromosome 1 in line IV (Fig. 1) of the Monte Baldo population (Winterfeld 11) are the result of a translocation from the homologous chromosome 11, which is devoid of subtelomeric heterochromatin and considerably shorter. As a result, the chromosomes of this pair are par II. In both populations only one of the four SAT chromosomes of *H. parlatorei* has subtelomeric DAPI bands in the arm without the satellite. Modifications of both karyotypes were found in the different number of eight (Petzen population) and 13 (Monte Baldo population) DAPI heterochromatin bands which correlate partially with the satellite DNAs CON1 and CON2. Based on the hypothesis of heterochromatin accumulation during evolution (Guerra *et al.* 2000; Ikeda 1988; Morawetz and Samuel 1989; Röser 1994; Winterfeld and Röser 2007b), the population of *H. parlatorei* from Monte Baldo might be considered younger.

In contrast to *H. parlatorei*, both subspecies of *H. setaceum* were characterized by subtelomeric CON1 and CON2 satellite DNA and DAPI bands on both arms of the 5S rDNA-bearing chromosomes, termed ‘marker chromosomes set’ (pink square in Fig. 1), making these chromosomes distinguishable from those of *H. parlatorei*. However, in addition to the varying number of satellite chromosomes (three in subsp. *setaceum* and four in subsp. *petzense*), the subspecies differ in the number of 5S rDNA bands (two in subsp. *setaceum* and three in subsp. *petzense*) and the number of DAPI-positive heterochromatin bands and their localization (15 in subsp. *petzense* and 17 in subsp. *setaceum*). All in all, chromosomal characters of subsp. *setaceum* seem to be derived from subsp. *petzense* by loss of one NOR (cf. Winterfeld and Röser 2007a) and 5S rDNA sequences (cf. Wendel *et al.* 1995) or their inactivation (cf. Chen and Pikaard 1997; Pikaard, 1999) and by changes in the DAPI heterochromatin banding pattern (cf. Guerra *et al.* 2000; see Fig. 1).

The presumed hybrid plants of *H.* ×*krischae* and *H.* cf. ×*krischae* investigated here were found to be diploid like their parents (cf. Sauer 1971). Their karyotypes contain marker chromosomes (set, par I, par II) as well as satellite chromosomes from both *H. parlatorei* and *H. setaceum*. Therefore, at the chromosome level, they are intermediate between their presumable parental taxa. Their common geographical distribution as well as co-possession of B chromosomes in *H.* ×*krischae* and *H. setaceum* subsp. *petzense* supports the latter as one parent of the hybrid *H.* ×*krischae*. This is in agreement with intermediate morphological characters, especially noted in the laminas of *H.* x*krischae* leaves (Heuchert and Röser 2004; Melzer 1967).

Interestingly, the localization of DAPI positive heterochromatin and satellite DNA CON1 and CON2 bands in the chromosomes of *H.* ×*krischae* and *H.* cf. ×*krischae* are not fully intermediate between their parents. Obviously either translocations between single chromosomes of the parental taxa occurred, or these hybrid plants are not immediate F_1_ hybrids, indicating that other intermediates between *H. parlatorei* and *H. setaceum* subsp. *petzense* occur. It appears the examined plants belong to a complex hybrid swarm in which the parental plants are in a continuous process of crossing and backcrossing due to the lack of a barrier to gene flow. This result supports the morphometric findings (Heuchert and Röser 2004), in which hybrid plants display a mosaic of characters of their parental taxa characteristic of many other natural hybrid plants (Arnold 1997; Rieseberg and Ellstrand 1993).

The hexaploid cytotype of *H. sempervirens* shares the karyotype and the marker chromosomes par I with diploid *H. parlatorei*, especially of the population from Monte Baldo, but is missing the characteristic chromosome pattern of *H. setaceum*, which would be expected based on the Topo6 data. Which subspecies of *H. setaceum* is the second parent of *H. sempervirens* cannot be determined from the molecular and cytogenetic methods used, because the Topo6 sequences of subsp. *petzense* and subsp. *setaceum* are highly similar and their chromosomal differences, which are widely restricted to subtelomeric heterochromatin bands, are rather weak. The close relationship of *H. sempervirens* to the diploids *H. parlatorei* and *H. setaceum*, which was taxonomically implied (Röser 1989, 1996), is corroborated by molecular data. Deviations in the karyotype of *H. sempervirens* which shows a reduction of 5S rDNA loci, because the number of 5S rDNA bands are not a multiple of their alleged diploid parental taxa, is presumably caused by unidirectional homogenization consistently at the cost of repeats from the *H. setaceum* genome. This result represents an example of a beginning diploidization in the course of polyploidization, found also in other plant groups (e.g. Ma and Gustafson 2005; Ozkan and Feldman 2009; Renny-Byfield *et al.* 2013).

#### *Biogeography and evolution of the* H. parlatorei *group*

The current occurrence of the alpine *H. parlatorei* group seems to be influenced by glaciations during the last ice age period: the distribution of widespread *H. parlatorei*, known mostly from the southern calcareous flank of the Alps (Pignatti 1982) and minor refuge areas in the Bavarian and Austrian Alps of the northern flank, lies in regions of the Alps that remained unglaciated during the last glacial period (cf. Merxmüller 1952; Pawlowski 1970; Schönswetter *et al.* 2005). The disjunct east-west geographic occurrence of the two subspecies of *H. setaceum* implies that their distribution was also shaped during glacial periods, with the southwestern and southeastern Alps serving as important refuge areas (cf. Merxmüller 1952).

Cytogenetic evidence, e.g. the presumably older karyotype of *H. setaceum* subsp. *petzense* (see above), points to a primary center of speciation in the eastern region of the Alps. Processes of hybridization with *H. parlatorei* likely took place when the parental taxa became sympatric. As a result, the homoploid hybrid *H.* ×*krischae* and backcross offspring with the parents (*H.* cf. ×*krischae*) have become established on the Petzen Mountain.

**Figure 3.**
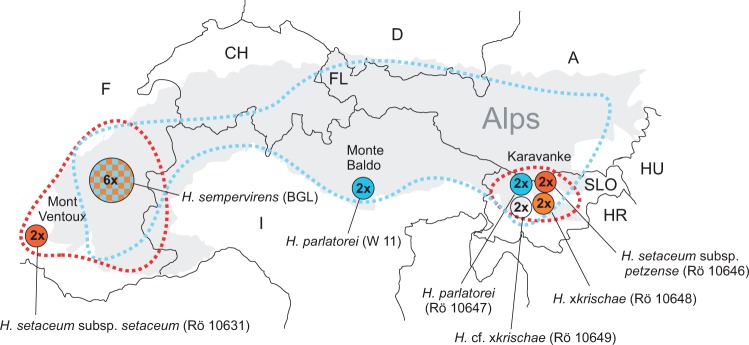
Collection sites of studied taxa of the *H. parlatorei* group in the Alps and simplified general geographical distribution of *H. parlatorei* (blue dotted line) and disjunct areas of *H. setaceum* subsp. *setaceum* in the West and subsp. *petzense* in the East (red dotted lines). Circles filled with checkerboard pattern indicate hybrid character in molecular and cytogenetic analyses.

Westward expansion of this diploid hybrid (or hybrid swarm) initially resulted in a large southern alpine distribution area. Most probably during the ice age glaciations this range became fragmented into two distinct areas in the eastern and the western Alps with a gap in distribution of ca. 600 km. It is possible that in the western area harboring currently *H. setaceum* subsp. *setaceum* and *H. sempervirens* (whose genome also indicates a hybrid origin), polyploidization took place in the latter species after isolation of the glacial refuges. Another hypothesis implies that hybridization took place at least two times in parallel in the western and the eastern Alps followed by polyploidization only in western *H. sempervirens* and genome diploidization (Fig. 3).

Recently, the hybrid plants *H.* ×*krischae* and *H.* cf. ×*krischae* but also the polyploid *H. sempervirens* have narrower overall geographical distributions than their putative diploid parents *H. parlatorei* and *H. setaceum* (see Fig. 3). This is a clear example where genetic introgression by homoploid hybridization on the one hand and maybe followed by polyploidization on the other hand of more widely distributed diploid species like *H. parlatorei* and/or *H. setaceum* resulted in taxa with smaller distribution areas. The same pattern has been reported in other polyploid series of other species, e.g. of the genus *Helictochloa* (= *Helictotrichon*; cf. Winterfeld *et al.* 2009b), which was discussed in relation to the allopolyploid origin of these taxa. This contrasts to the general opinion, that polyploid plants are more vital and efficient colonizers than their diploid ancestors.

## Conclusions

The cytogenetic and molecular phylogenetic data confirm that the endemic Karavanke Range *H.* ×*krischae* and *H.* cf. ×*krischae* in the southeastern Alps are homoploid hybrids, resulting from a cross between the widespread alpine *H. parlatorei* and the local endemic *H. setaceum* subsp. *petzense*. Hexaploid *H. sempervirens* from the southwestern Alps, the French Alps and their Préalps is also suspected to be of hybrid origin involving the *H. parlatorei* and *H. setaceum* genomes. Cytogenetic evidence, e.g. the potentially older karyotype of *H. setaceum* subsp. *petzense*, might suggest a primary center of species establishment in the eastern regions of the Alps, with the westward expansion and subsequent ice age interruption resulting in a recently disjunct distribution between populations of the southwestern and southeastern Alps.

## Contributions by the Authors

G.W.: molecular cytogenetic analyses, manuscript preparation; A.W.: molecular phylogenetic analyses; M.R.: proofreading.

## Conflicts of Interest

None declared.
